# Klinische Wirksamkeit der aurikulären Vagusnervstimulation in der Behandlung chronischer und akuter Schmerzen: Eine systematische Übersichtsarbeit

**DOI:** 10.1007/s00482-022-00686-2

**Published:** 2023-01-02

**Authors:** Rudolf Likar, Christophe Perruchoud, Stefan Kampusch, Markus Köstenberger, Sabine Sator, Caroline Stremnitzer, Andreas Wolf, Stefan Neuwersch-Sommeregger

**Affiliations:** 1 Abteilung für Anästhesiologie und Intensivmedizin, Klinikum Klagenfurt am Wörthersee, Feschnigstraße 11, 9020 Klagenfurt, Österreich; 2 Paracelsus Medizinische Privatuniversität, Salzburg, Österreich; 3 Clinique de la Douleur, Hopital de La Tour, Genf, Schweiz; 4 AURIMOD GmbH, Wien, Österreich; 5 Universitätsklinik für Anästhesie, allgemeine Intensivmedizin und Schmerztherapie, Medizinische Universität Wien, Wien, Österreich; 6 Abteilung für Anästhesie, Krankenhaus St. Vinzenz Zams, Zams, Österreich; 7 Medizinische Universität Graz, Graz, Österreich

**Keywords:** Neuromodulation, Minimal invasive Schmerztherapie, Chronische Kreuzschmerzen, Chronische Migräne, Postoperative Schmerzen, Neuromodulation, Minimal invasive pain therapy, Chronic low back pain, Chronic migraine, Postoperative pain

## Abstract

**Hintergrund:**

Aktuelle Leitlinien empfehlen für die Behandlung chronischer Schmerzen einen personalisierten, multimodalen und interdisziplinären Ansatz. Bereits in der akuten Behandlung postoperativer Schmerzen kann es sinnvoll sein, Risikofaktoren für die Chronifizierung zu minimieren. Die aurikuläre Vagusnervstimulation (aVNS) könnte eine effektive nichtmedikamentöse Therapie zur Behandlung von Schmerzen darstellen.

**Ziel der Arbeit:**

Ziel dieser Arbeit ist die Evaluierung der klinischen Wirksamkeit der aVNS bei chronischen und akuten Schmerzen sowie deren Einfluss auf die begleitende Medikamenteneinnahme.

**Material und Methoden:**

Es wurde eine systematische Literaturrecherche zur Anwendung aurikulärer elektrischer Stimulation bei chronischen und akuten Schmerzen durchgeführt. Die Studien wurden entsprechend ihrem Evidenzgrad klassifiziert, gemäß Jadad-Skala und wissenschaftlicher Validität bewertet, und anschließend in Bezug auf Indikation, Methode, Stimulationsparameter, Behandlungsdauer, Wirksamkeit und Sicherheit analysiert.

**Ergebnisse:**

Es konnten 20 Studien zu chronischen Schmerzindikationen, 10 Studien zu akuten postoperativen Schmerzen sowie 7 Studien zu experimentellen akuten Schmerzen identifiziert und analysiert werden. Die Recherche ergab eine Gesamtanzahl von *n* = 1105 mit aVNS behandelten Patient*innen. Die beste Evidenz zur Wirksamkeit der aVNS liegt für die Indikationen chronischer Kreuzschmerz, chronisches Zervikalsyndrom, chronischer Unterleibsschmerz und chronischer Migräne sowie zu akutem postoperativen Schmerz bei Oozytenaspiration, laparoskopischer Nephrektomie und offenen kolorektalen Eingriffen vor. In der Mehrzahl der Studien konnte außerdem eine signifikante Reduktion der Schmerzmittel- bzw. Opiateinnahme gezeigt werden. In 3 randomisierten kontrollierten Studien bei chronischen Schmerzpatient*innen konnte eine nachhaltige Schmerzreduktion über einen Zeitraum von bis zu 12 Monaten gezeigt werden. Insgesamt wurde die aVNS sehr gut vertragen.

**Schlussfolgerung:**

Die Studienlage zeigt, dass die aVNS eine ergänzende, effektive nichtmedikamentöse Behandlung für Patient*innen mit chronischen und akuten postoperativen Schmerzen sein kann. Zukünftige Studien in den genannten Indikationen sollten auf eine Standardisierung und Optimierung von Behandlungsparametern, die stärkere Einbeziehung von Quality-of-Life-Outcome-Parametern sowie längere Follow-up-Perioden zum besseren Verständnis der nachhaltigen therapeutischen Wirkung der aVNS fokussieren.

**Zusatzmaterial online:**

Die Online-Version dieses Beitrags (10.1007/s00482-022-00686-2) enthält eine Tabelle mit einer ausführlichen Zusammenfassung und Analyse der Studien.

## Abstract (en)

**Background:**

Current guidelines recommend a personalized, multimodal, and interdisciplinary approach for the treatment of chronic pain. Already in the acute treatment of postoperative pain, it can be useful to minimize risk factors for chronification. Auricular vagus nerve stimulation (aVNS) could be an effective non-drug therapy for the treatment of chronic and acute pain.

**Aim of the work:**

The aim of this systematic review is to evaluate the clinical efficacy of aVNS in chronic and acute pain as well as its effect on medication intake.

**Materials and methods:**

A systematic literature search was carried out on the application of auricular electrical stimulation in chronic and acute pain. Studies were classified according to their level of evidence and evaluated via the Jadad scale as well as their scientific validity, and then analyzed in terms of indication, method, stimulation parameters, duration of treatment, efficacy, and safety.

**Results:**

Twenty studies on chronic pain indications, ten studies on acute postoperative pain, as well as seven studies on experimental acute pain were identified and analyzed. The search revealed a total of *n* = 1105 aVNS-treated patients. The best evidence on the efficacy of aVNS is available for the indications chronic low back pain, chronic cervical syndrome, chronic abdominal pain, and chronic migraine as well as acute postoperative pain in oocyte aspiration, laparoscopic nephrectomy, and open colorectal surgery. Additionally a significant reduction in analgesic or opiate intake was evident in most studies. In three randomized controlled trials in chronic pain patients, a sustainable pain reduction over a period of up to 12 months was shown. Overall, aVNS was very well tolerated.

**Conclusion:**

This review indicates that aVNS can be a complementary and effective non-drug treatment for patients with chronic and acute postoperative pain. Future studies in these indications should focus on standardizing and optimizing treatment parameters, inclusion of quality-of-life outcome parameters, and longer follow-up periods to better understand the sustainable therapeutic effect of aVNS.

## Einleitung

Chronische Schmerzen betreffen mehr als 30 % der Menschen weltweit und verursachen oft eine massive Einschränkung der Lebensqualität sowie hohe sozioökonomische Kosten [16]. Insbesondere Rückenschmerzen, Kopf- und muskuloskelettale Schmerzen zählen zu den Erkrankungen mit den höchsten „years lost to disability“ (YLD) [16].

Die Behandlung chronischer Schmerzpatient*innen ist komplex. Aktuelle Leitlinien empfehlen einen personalisierten, multimodalen und interdisziplinären Ansatz sowie den Einsatz medikamentöser und nichtmedikamentöser Therapien [16]. Als Beispiel wird bei chronischem unspezifischem Kreuzschmerz eine Erstlinientherapie mit Acetaminophen und nichtsteroidalen entzündungshemmenden Wirkstoffen (NSAIDs) empfohlen, gefolgt von kurzzeitigem Einsatz von Muskelrelaxanzien oder Opioid-Analgetika und in Kombination mit multidisziplinärer Rehabilitation, kognitiver Verhaltenstherapie oder neuromodulativen Verfahren [6, 21, 63].

Bereits in der akuten Behandlung postoperativer Schmerzen kann es sinnvoll sein, Risikofaktoren für die Chronifizierung dieser Schmerzen (u. a. durch periphere oder zentrale Sensibilisierung) zu minimieren [28]. Es gilt zu prüfen, ob zum Beispiel neuromodulative Verfahren eine effektive und ergänzende nichtmedikamentöse Therapie zur Reduzierung postoperativer Schmerzen darstellen können und dadurch das Risiko für chronische Schmerzen reduziert werden kann.

Mit den vorhandenen pharmakologischen Therapien werden teilweise nur geringe und/oder kurzfristige Verbesserungen erzielt. Nebenwirkungen und/oder Wechselwirkungen mit anderen medikamentösen Therapien müssen berücksichtigt und eine mögliche Abhängigkeit bei Langzeitanwendung von Opioiden als Risiko abgewogen werden. Der aktuelle wissenschaftliche Fokus liegt daher auf der Erforschung ergänzender, effektiver und sicherer nichtmedikamentöser Behandlungsoptionen und insbesondere neuromodulativer Ansätze [63].

### Der Vagusnerv und Schmerz

Der Vagusnerv ist der zehnte und längste von insgesamt zwölf Hirnnerven und der wichtigste parasympathische Nerv im autonomen Nervensystem [37, 38]. Er nimmt seinen Ursprung in der Medulla oblangata im Hirnstamm und tritt im Bereich der Medulla, gemeinsam mit dem Nervus accessorius und dem Nervus glossopharyngeus, an die Hirnoberfläche, zieht durch das Foramen jugulare aus dem Schädel und innerviert Hals- und Rachenraum, Thorax und Abdomen sowohl sensorisch als auch motorisch und parasympathisch.

Die weitreichenden Projektionen des Vagusnerv sind wesentlich für die Gehirn-Körper-Interaktion und die Aufrechterhaltung der autonomen Funktionen. Etwa 80 % der vagalen Fasern sind afferent. Sie nehmen eine Vielzahl an sensorischen Reizen aus der Peripherie auf (Mechano‑, Thermo‑, Nozizeption) und leiten diese an die vagalen Hirnstammkerne Nucleus spinalis nervi trigemini (NSNT) und Nucleus tractus solitarii (NTS) weiter [37, 38].

**Abb. 1:**
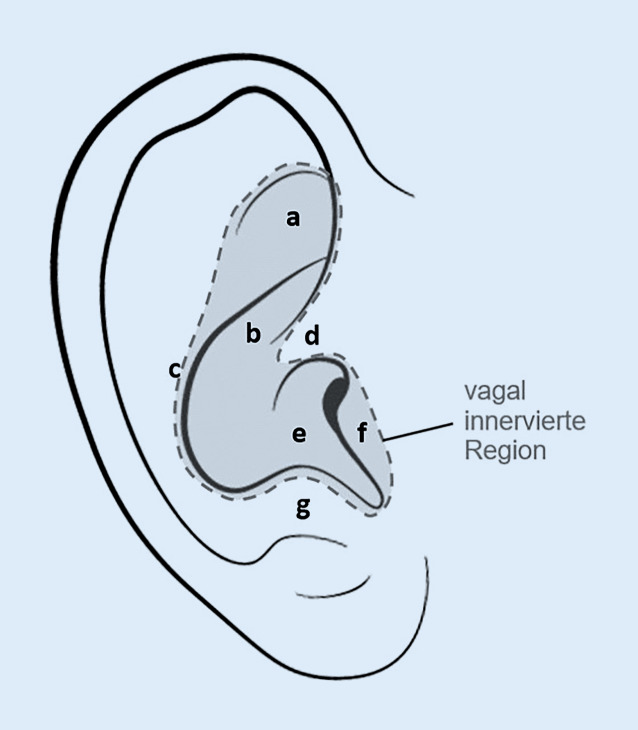
Ohranatomie mit eingezeichnetem vagal innervierten Bereich und spezifischen anatomischen Regionen [12, 55]. *a* Fossa triangularis, *b* Cymba conchae, *c* Anthelix, *d* Crus helicis, *e* Cavum conchae, *f* Tragus, *g* Antitragus

Über seinen aurikulären Ast innerviert der Vagusnerv auch das Außenohr sensorisch [12]. Somit ist der Vagusnerv über die Haut der Ohrmuschel in den Bereichen der Cymba conchae, der Concha, sowie in geringerem Ausmaß der (Crus) Anthelix, der Fossa triangularis, des Tragus und der Crus helicis zugängig (siehe Abb. 1; [12, 55]). Nachweise der afferenten vagalen Innervierung des Ohrs sowie der Projektion zu NSNT und NTS konnten in Tracer-Studien im Tierversuch, durch anatomische Studien im Menschen und auch durch funktionelle Magnetresonanzuntersuchungen erbracht werden [12, 18, 68, 81].

Ob über die vagalen Afferenzen die Nozizeption moduliert werden kann, wurde erstmals in systematischen Studien in den 1980er Jahren untersucht [3, 4, 58, 72, 73]. Die peripheren und zentralen Systeme, die zum Beispiel kardiovaskuläre und autonome Funktionen regulieren, wurden dort als eng verbunden mit den in die Kontrolle von Nozizeption involvierten Systemen beschrieben [59]. Mittlerweile konnte gezeigt werden, dass über die afferente Stimulation von NTS und NSNT eine Vielzahl relevanter Gehirnstrukturen moduliert wird, wie u. a. Nucleus dorsalis nervi vagi, Locus ceruleus (noradrenerg), Raphe-Nuclei (RN; serotonerg), Amygdala, Thalamus, periaquäduktales Grau (PAG), cingulärer Cortex, präfrontaler Cortex [13, 38, 68].

Verarbeitung und Wahrnehmung von Schmerz erfolgen kurz zusammengefasst auf der Ebene des Hirnstamms (Formatio reticularis inkl. RN) zur Steuerung autonomer Prozesse, des Thalamus als Relay zum Großhirn und anderen Hirnregionen, der Großhirnrinde zur Steuerung der Wahrnehmung und Bewusstwerdung von Schmerz, des Hypothalamus zur Regulation der hormonellen Antwort und des limbischen Systems zur Emotionsbelegung von Schmerz [13, 37, 38, 59, 68, 84].

Eine analgetische Wirkung der aurikulären Vagusnervstimulation (aVNS) kann nach heutigem Wissensstand auf folgende Mechanismen zurückgeführt werden: (1) Eine teilweise Aktivierung absteigender noradrenerger und serotonerger Systeme verbunden mit einer Ausschüttung von Enkephalin und einer entsprechenden Wirkung auf Opioid Rezeptoren [38, 68], (2) eine Wirkung auf das limbische System, wie z. B. bei Migränepatient*innen gezeigt [13, 26, 84], (3) eine parasympathische Aktivierung und sympatholytische Wirkung [26, 37] sowie (4) der sog. vagal mediierte cholinerge antiinflammatorische Reflex können positiv auf das Schmerzgeschehen bzw. die Schmerzreduktion wirken [37, 57, 80].

### Aurikuläre Vagusnervstimulation

Die elektrische Stimulation des zervikalen Astes des Vagusnervs ist seit den 1990er Jahren zur Behandlung von therapierefraktärer Epilepsie und chronischer, therapieresistenter Depression zugelassen [37]. Bei dieser Art der Vagusnervstimulation wird ein Impulsgenerator implantiert, der über eine Cuff-Elektrode elektrische Impulse an den zervikalen Vagusnerv (präferiert linksseitig) abgibt.

Neuere Studien beschäftigen sich mit nicht- oder minimal-invasiven Technologien zur Vagusnervstimulation, um Nebenwirkungen (z. B. Heiserkeit, Husten, Schmerzen oder Schluckbeschwerden durch unspezifische afferente und efferente Stimulation), Risiken der Implantation und Kosten zu reduzieren. Weiters soll die Methode in einem breiteren Indikationsspektrum und einer größeren Patient*innengruppe einsetzbar werden [37, 83]. Diese Ansätze basieren entweder auf der transkutanen Stimulation des zervikalen Vagusnervs mittels Oberflächenelektroden oder auf der transkutanen/perkutanen Stimulation des aurikulären Vagus Nervs mittels Oberflächen- oder Nadelelektroden [23, 82]. Solche nicht- oder minimal-invasiven Verfahren werden aktuell bereits in der Behandlung von Epilepsie, Depression, chronischen Rückenschmerzen, Migräne oder postoperativen Schmerzen eingesetzt. Potenzielle therapeutische Effekte werden derzeit auch in einer Vielzahl an Studien in weiteren Indikationen untersucht (u. a. Tinnitus, entzündliche Erkrankungen oder Schlaganfallrehabilitation), wie in [23, 82, 83] beschrieben.

Umfassende Übersichtsarbeiten zum Einsatz der aVNS bei chronischen und akuten Schmerzpatient*innen fehlen. Ziel dieser systematischen Übersichtsarbeit ist die Zusammenfassung, Evaluierung und Analyse vorhandener Studiendaten zu Anwendung, Wirksamkeit und Sicherheit der aVNS bei akutem und chronischem Schmerz.

## Material und Methoden

Eine systematische Literaturrecherche mit Inhaltsanalyse wurde in den Datenbanken PubMed, Scopus und Semantic Scholar durchgeführt. Die Suche wurde auf den Zeitraum 01.01.2000 bis 01.06.2022 begrenzt und alle Datenbanken mit festgelegten, thematisch relevanten Schlagwörtern wurden durchsucht. Folgende Schlagwörter wurden festgelegt: auric* vagus nerve stimulation, auric* elect* stimulation, auric* elect* vagus nerve stimulation, auricular neurostimulation (+ pain [bei > 1000 Ergebnissen]), VNS and pain.

Die Ergebnisse wurden nach Duplikaten durchsucht und diese fallweise entfernt. Die verbleibenden Arbeiten wurden auf Basis von Titel, Abstract und folgenden Ausschlusskriterien gescreent: Publikationsjahr < 2000, kein Abstract vorhanden, verwendete Sprache nicht Englisch oder Deutsch, präklinische Studie/Tierstudie, Studienprotokolle, Fallstudie, Review, kein Bezug zu aVNS. Zur weiteren Qualifikation der Studien wurde anhand des Volltexts auf die Übereinstimmung mit den Einschlusskriterien Indikation (Schmerz) und Intervention (aVNS, transkutan oder perkutan) geprüft.

Die eingeschlossenen Studien wurden von zwei unabhängigen Reviewern mittels der Jadad-Skala (max. Punktezahl 5) und entsprechend ihrer wissenschaftlichen Validität (max. Punktezahl 4) bewertet (Tab. 1) und die jeweiligen Mittelwerte der Bewertungen summiert (mögliche Gesamtpunktezahl 9) [32]. Studien, die nicht nach der Jadad-Skala beurteilt werden konnten (keine Randomisierung, keine Verblindung), wurden nur anhand der festgelegten wissenschaftlichen Validitätskriterien bewertet (max. 4 Punkte) [2, 29, 56].

**Tab. 1:** Jadad-Skala (übersetzt nach [32]) und Evaluierung der wissenschaftlichen Validität. (Erarbeitet nach [2, 29, 56])

*Jadad-Skala*			
Wurde die Studie als randomisiert angegeben?		Ja/Nein	+1/+0
Wurde die Studie als doppelblind angegeben?		Ja/Nein	+1/+0
Wurde das Ausscheiden von Probanden angemessen angeführt?		Ja/Nein	+1/+0
Wurde die Randomisierungsmethode beschrieben und war diese geeignet/angemessen?		Ja	+1
Wurde die Randomisierungsmethode beschrieben, war aber ungeeignet?		Ja	−1
Wurde die Verblindungsmethode beschrieben und war diese geeignet/angemessen?		Ja	+1
Wurde die Verblindungsmethode beschrieben, war aber ungeeignet?		Ja	−1
*Evaluierung der wissenschaftlichen Validität*			
Endpunkte	Reflektiert die Wahl und Auswertung der Endpunkte das relevante Indikationsspektrum?	Ja/Nein	+1/+0
Follow-up/Anwendungsdauer	Ist die gewählte Follow-up-Periode ausreichend lange, um zu beurteilen, ob die Anwendungsdauer Einfluss auf Outcome/Komplikationen hat bzw. Komplikationen identifiziert werden können?	Ja/Nein	+1/+0
Statistische Methodik	Wurde eine angemessene statistische Analyse der Daten durchgeführt und angegeben?	Ja/Nein	+1/+0
Klinische Signifikanz	War die beobachtete Wirksamkeit der Behandlung klinisch/statistisch signifikant?	Ja/Nein	+1/+0

Die Volltexte der Publikationen wurden analysiert und zusammengefasst in Bezug auf Studientyp und Evidenzgrad [8, 29], Indikation (Klassifikation in: chronischer Schmerz, akuter postoperativer Schmerz, akuter experimenteller Schmerz), Methode (Intervention, Stimulationspunkte – siehe auch Abb. 1 – und Kontrolle), Stimulationsparameter, Behandlungsdauer, primäre und sekundäre Ergebnisse sowie beobachtete unerwünschte Ereignisse.

## Ergebnisse

**Abb. 2:**
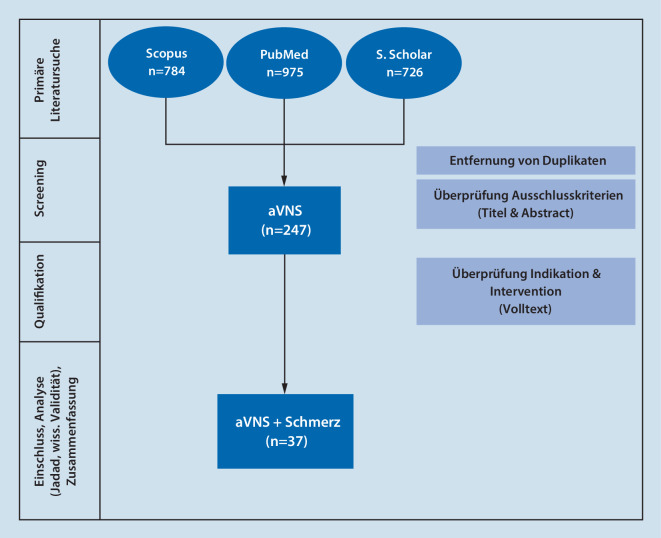
Flussdiagramm und Ergebnisse der systematischen Literaturrecherche. *aVNS* aurikuläre Vagusnervstimulation

**Abb. 3:**
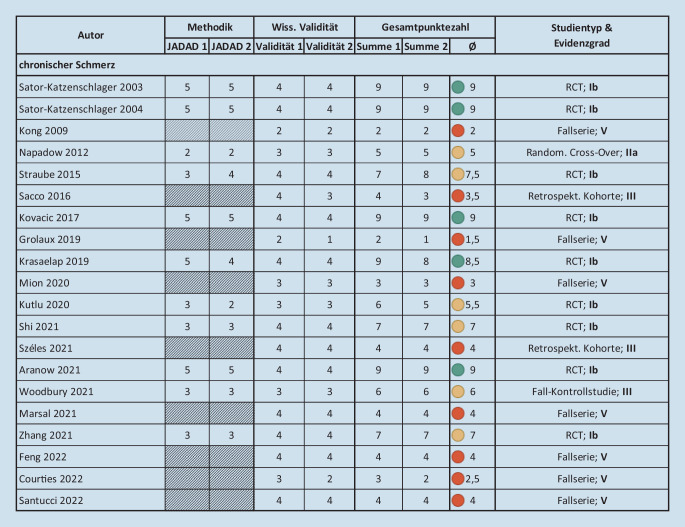
Ergebnisse der Bewertung der Qualität sowie des Evidenzgrads der eingeschlossenen Studien – chronischer Schmerz. *RCT* randomisierte kontrollierte Studie

Insgesamt wurden bei der primären Literatursuche *n* = 2485 Ergebnisse (Abb. 2) gefunden. Von diesen wurden nach dem Screening anhand Titel und Abstract *n* = 247 Studien qualifiziert. Nach einer weiteren Überprüfung wurden *n* = 37 Studien entsprechend der Jadad-Skala und der wissenschaftlichen Validität bewertet und in die weitere Analyse aufgenommen. Die Ergebnisse der Bewertung finden sich in den Abb. 3, 4 und 5, die Zusammenfassung und Analyse der Studien findet sich in Tab. 2 und im Online-Zusatzmaterial.

**Abb. 4:**
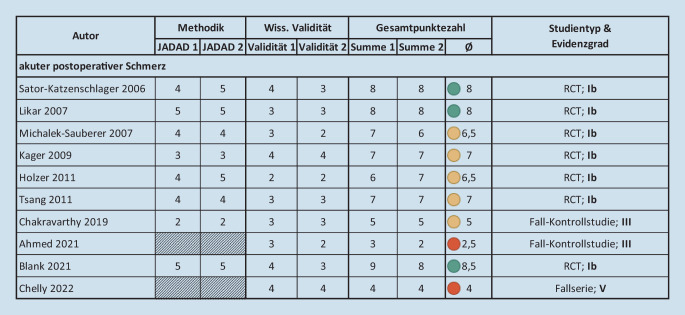
Ergebnisse der Bewertung der Qualität sowie des Evidenzgrads der eingeschlossenen Studien – akuter postoperativer Schmerz. *RCT* randomisierte kontrollierte Studie

**Abb. 5:**
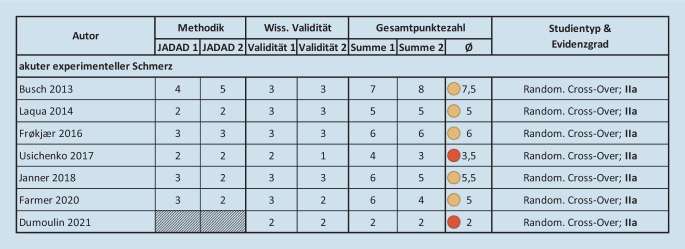
Ergebnisse der Bewertung der Qualität sowie des Evidenzgrads der eingeschlossenen Studien – akuter experimenteller Schmerz

**Tab. 2:** Zusammenfassung und Analyse der Studien zur Anwendung der aVNS bei chronischen Schmerzen, akuten postoperativen Schmerzen und akuten experimentellen Schmerzen – Kurzversion

Autor	Indikation	Primäres Ergebnis
*Chronischer Schmerz*		
Sator-Katzenschlager 2003 [66]	Chronisches Zervikalsyndrom	***VAS*** **↓** pVNS vs. Sham
Sator-Katzenschlager 2004 [65]	Chronischer Kreuzschmerz	***VAS*** **↓** pVNS vs. Sham
Kong 2009 [39]	Spondylose, Migräne	*VAS* ↓ pVNS vs. Ausgangswert
Napadow 2012 [53]	Chronischer Beckenschmerz/Endometriose	Evozierte Schmerzintensität ↓ tVNS vs. Sham
Straube 2015 [70]	Chronische Migräne	***Kopfschmerztage ↓*** tVNS (1 Hz) vs. tVNS (25 Hz)
Sacco 2016 [62]	Chemotherapie-induzierte periph. Neuropathie	***NRS ↓*** pVNS vs. Ausgangswert
Kovacic 2017 [40]	Chronischer Unterleibschmerz (11–18 Jahre)	***PFSD ↓*** pVNS vs. Sham
Grolaux 2019 [30]	IB-Schmerz und chronischer Schmerz	IBS-SSS ↓ tVNS vs. Ausgangswert
Krasaelap 2019 [41]	IBD-Schmerz	***Schwere abdominale Schmerzen ↓*** pVNS vs. Ausgangswert
Mion 2020 [49]	IBS-Schmerz	***IBS-SSS*** **↓** tVNS vs. Ausgangswert
Kutlu 2020 [43]	Fibromyalgie	VAS ↓, Depression ↓, Angst ↓, Funktionalität ↑, SF-36 ↑ tVNS + Training vs. Training
Shi 2021 [69]	IBS‑C, chronischer abdominaler Schmerz	***CSBMs/Woche*** **↑**, ***VAS*** **↓** tVNS vs. Sham
Széles 2021 [71]	Chronischer Rückenschmerz	***NRS*** **↓** pVNS vs. Ausgangswert
Aranow 2021 [5]	SLE und muskuloskelettaler Schmerz	***Schmerz*** **↓**, ***Müdigkeit ↓*** tVNS vs. Sham
Woodbury 2021 [79]	Fibromyalgie	VAS ↓, ***Schlaf*** **↑*, Aktivität ↑, Stimmung ↑*** pVNS vs. Kontrolle
Marsal 2021 [46]	Rheumatoide Arthritis	***DAS28-CRP*** **↓** tVNS vs. Ausgangswert
Zhang 2021 [84]	Migräne ohne Aura	***Migränetage*** **↓,** ***Schmerzintensität*** **↓**, ***Dauer ↓ ***tVNS vs. Sham
Feng 2022 [24]	Migräne ohne Aura	***VAS ↓,*** ***Attackenanzahl ↓, Dauer ↓, MSQ*** **↑**, ***SDS ↓, SAS*** **↓ **tVNS vs. Ausgangswert
Courties 2022 [17]	Osteoarthritis/Handschmerz	***VAS*** **↓** tVNS vs. Ausgangswert
Santucci 2022 [64]	Chron. funktioneller Unterleibschmerz (11–18 J.)	***VAS*** **↓*, Übelkeit*** **↓, *Angst* ↓** pVNS vs. Ausgangswert
*Akuter postoperativer Schmerz*		
Sator-Katzenschlager 2006 [67]	Perioperativ (Oozytenaspiration)	***VAS*** **↓** pVNS vs. Sham
Likar 2007 [45]	Postoperativ (laparoskopische Nephrektomie)	***Ruhe-VAS*** **↓,** ***Belastungs-VAS*** **↓** 1. Stunde post-OP pVNS vs. Sham
Michalek-Sauberer 2007 [48]	Postoperativ (Backenzahn-Extraktion)	VAS ↔, Analgetikabedarf ↔ pVNS vs. Sham
Kager 2009 [35]	Postoperativ (Tonsillektomie)	***VAS*** **↓ **9, 12, 24 h postoperativ pVNS vs. Sham
Holzer 2011 [31]	Postoperativ (gynäkologische Eingriffe)	VAS ↔ pVNS vs. Sham
Tsang 2011 [74]	Postoperativ (Hysterektomie)	***VAS*** **↓**, PEFR ↔ tVNS vs. Ausgangswert/Kontrolle
Chakravarthy 2019 [14]	Postoperativ (Sectio)	***NRS*** **↓ **pVNS vs. Kontrolle
Ahmed 2021 [1]	Postoperativ (Roux-en‑Y Magen-Bypass)	OME **↓ **24 h post-OP pVNS vs. Kontrolle
Blank 2021 [7]	Postoperativ (kolorektale Eingriffe)	OME ↔ pVNS vs. Sham (gesamt); ***OME*** **↓ **pVNS vs. Sham (nach offenem Eingriff)
Chelly 2022 [15]	Postoperativ (Nierenspende)	***OME* ↓** 24 h post-OP pVNS vs. Kontrolle
Akuter experimenteller Schmerz in gesunden Proband*innen		
Busch 2013 [11]	Experimenteller mechan./Hitzeschmerz	Schmerzschwelle ↑ mechan./Druckschmerz, ***Schmerzen* ↓** ***(Hitze)*** tVNS vs. Sham
Laqua 2014 [44]	Experimentelle Schmerzschwelle	***Schmerzschwelle*** **↑** (*n* = 15),*** Schmerzschwelle*** **↓**(*n* = 6) tVNS vs. Kontrolle
Frøkjær 2016 [27]	Akuter mechan. Schmerz, Darmmotilität	CPM ↔,*** Schmerzschwelle Knochenschmerz*** **↑ **tVNS vs. Sham
Usichenko 2017 [75]	Experimenteller Hitzeschmerz	***Schmerzschwelle*** **↑** (*n* = 8),*** Schmerzschwelle*** **↓**(*n* = 12) tVNS vs. Sham
Janner 2018 [33]	Experimenteller Hitzeschmerz	***VAS*** **↓** tVNS vs. Kontrolle
Farmer 2020 [22]	Ösophagaler Schmerz, Hypersensibilität (HS)	***Verhinderte/reversierte*** Säure-induzierte ösophagale HS tVNS vs. Sham
Dumoulin 2021 [19]	Experimenteller Schmerz	Somatosensorische Perzeption ↔ tVNS vs. Sham

*CPM* Conditioned Pain Modulation, *CSBMs* Complete Spontaneous Bowel Movements, *DAS28-CRP* Disease Activity Score-28 with C-Reactive Protein, *IB* Irritable Bowel, *IBD* Irritable Bowel Disease, *IBS* Irritable Bowel Syndrome, *IBS-SSS* Irritable Bowel Syndrome-Severity Scoring System, *MSQ* Migraine-specific QoL Questionnaire, *NRS* Numeric Rating Scale, *OME* Oral Morphine Equivalents, *PEFR* Peak Expiratory Flow Rate, *PFSD* Pain Frequency-Severity-Duration, *pVNS* perkutane VNS, *SAS/SDS* self-rating anxiety scale/self-rating depression scale, *SLE* systemischer Lupus erythematodes, *tVNS* transkutane VNS, *VAS* Visuelle Analogskala, *VNS* Vagusnervstimulation, *↔* kein signifikanter Unterschied/keine Veränderung, *↑/↓* nicht signifikante Veränderung; *↑/↓* signifikante Veränderung dargestellt in ***fettkursiv***

### Analyse chronischer Schmerz

20 Studien zu chronischen Schmerzen mit einer Gesamtzahl an *n* = 633 aVNS-behandelten Patient*innen wurden in die Analyse aufgenommen. Diese Studien behandelten die Indikationen chronisch-entzündliche Darmerkrankungen/Unterleibschmerzen (7 Studien [30, 40, 41, 49, 53, 64, 69], *n* = 154), Migräne (4 Studien [24, 39, 70, 84], *n* = 133), Rückenschmerz (4 Studien [39, 65, 66, 71], *n* = 192), rheumatoide Arthritis (RA)/Osteoarthritis (2 Studien [17, 46]; *n* = 45), Fibromyalgie/systemischer Lupus Erythematodes (SLE) (3 Studien [5, 43, 79], *n* = 51), Chemotherapie-induzierte periphere Neuropathie (1 Studie [62]; *n* = 58), unspezifischer chronischer Schmerz (1 Studie [30], *n* = 3). Von diesen waren 9 Studien [5, 40, 41, 43, 65, 66, 69, 70, 84] randomisierte kontrollierte Studien (RCTs) hoher Qualität mit einer durchschnittlichen Bewertung von 7,5 aus 9 Punkten (Abb. 3). Eine Studie [53] war als randomisierte Cross-Over-Studie ausgelegt mit einer Bewertung von 5 Punkten. Des Weiteren wurden 7 Fallserien [17, 24, 30, 39, 46, 49, 64], 2 retrospektive Kohortenstudien [62, 71] und 1 Fall-Kontrollstudie [79] mit einer durchschnittlichen Bewertung von 3,1 Punkten identifiziert.

Schmerz auf einer Visuellen Analogskala (VAS) oder Numerischen Rating-Skala (NRS), psychisches Wohlbefinden und Verträglichkeit/Sicherheit der Stimulation waren die häufigsten primären und sekundären Endpunkte. Je nach Indikation wurden (zusätzliche) spezifischere symptombezogene und krankheitsrelevante Endpunkte gewählt.

In einer Mehrzahl der Studien konnte eine Verbesserung auf der VAS- oder NRS-Schmerzskala bzw. bei einem spezifischeren krankheitsbezogenen Endpunkt beobachtet werden, wobei diese Verbesserung in 16 Studien im Vergleich zum Ausgangswert und/oder im Vergleich zur gewählten Kontrollgruppe statistisch signifikant war (vgl. Tab. 2). In 3 der 9 RCTs (bei chronischem Rückenschmerz und Unterleibsschmerzen) konnte eine nachhaltige Schmerzreduktion über bis zu 12 Monate gezeigt werden [40, 65, 66].

In 8 Studien [17, 24, 49, 62, 65, 66, 70, 71] wurde der Bedarf an Schmerzmitteln erhoben. In 6 dieser Studien (davon 3 bei chronischem Rückenschmerz und 1 bei chronischer Migräne) [49, 62, 65, 66, 70, 71] konnte die Schmerzmitteleinnahme in der aVNS-Gruppe reduziert werden. Auch bei sekundären Endpunkten wie psychischem Wohlbefinden, Angst, Schlaf, Müdigkeit, Lebensqualität konnte – sofern erhoben – bei einer Mehrzahl der Studien eine signifikante Verbesserung erzielt werden [5, 24, 40, 43, 53, 64–66, 69, 70, 79].

Klinisch und statistisch hoch signifikante Ergebnisse konnten in den Indikationen chronischer Kreuzschmerz [65, 71], chronisches Zervikalsyndrom [66, 71], chronischer Unterleibsschmerz [40, 41, 69] und chronische Migräne [24, 70, 84] gezeigt werden.

Die aVNS wurde in den vorliegenden Studien typischerweise begleitend zu einer medikamentösen Therapie im Durchschnitt über einen Zeitraum von 5,84 Wochen (1–24 Wochen) eingesetzt. Die Behandlungsdauer sowie die Stimulationsparameter innerhalb dieser Anwendung waren jedoch teilweise sehr unterschiedlich.

In 3 Studien [24, 79, 84] wurden begleitend zur Therapie fMRI-Untersuchungen durchgeführt, um den Einfluss der aVNS auf die Gehirnaktivität näher zu charakterisieren. Dabei konnte im fMRI („functional magnetic resonance imaging“) insbesondere eine Verstärkung der Gehirnkonnektivität in den Bereichen des exekutiven Kontrollnetzwerks (präfrontale Gehirnregionen), des Thalamus und des Cerebellums unter bzw. kurz nach aVNS festgestellt werden.

### Analyse akuter postoperativer Schmerz

10 Studien zu akuten postoperativen Schmerzen mit insgesamt *n* = 246 aVNS-behandelten Patient*innen wurden in die Analyse aufgenommen. Die operativen Eingriffe betrafen gynäkologische Eingriffe (4 Studien [14, 31, 67, 74], *n* = 115), abdominale Eingriffe (4 Studien [1, 7, 15, 45], *n* = 67), Tonsillektomie (1 Studie [35], *n* = 16), und Backenzahnextraktion (1 Studie [48], *n* = 48). Von diesen waren 7 Studien [7, 31, 35, 45, 48, 67, 74] randomisierte kontrollierte Studien (RCTs) hoher Qualität mit einer durchschnittlichen Bewertung von 7,1 aus 9 Punkten (Abb. 4). 2 weitere Studien [1, 14] waren Fall-Kontrollstudien und 1 Studie [15] war eine Fallserie mit einer durchschnittlichen Bewertung von 3,8 Punkten.

7 Studien beobachteten eine signifikante Verbesserung in Bezug auf Schmerz und/oder Bedarf an Opioiden nach dem Eingriff unter Anwendung der aVNS, vgl. Tab. 2 [7, 14, 15, 35, 45, 67, 74]. In 1 Studie [1] wurden leichte, nichtsignifikante Verbesserungen beobachtet. Unterschiede in Bezug auf Übelkeit, Müdigkeit und Einnahme von Nicht-Opioid-Analgetika waren in keiner Studie signifikant (sofern erhoben).

Die aVNS wurde entweder kurz vor (perioperativ) oder direkt nach dem operativen Eingriff (postoperativ) für eine Dauer von 2–5 Tagen angewendet. Bei einer Studie (Hysterektomie) wurde nur wenige Minuten stimuliert [74], bei einer Studie (Oozytenaspiration) nur wenige Stunden [67].

Klinisch und statistisch hoch signifikante Ergebnisse konnten bei Oozytenaspiration zur In-vitro-Fertilisation [67], laparoskopischer Nephrektomie [45] sowie bei offenen kolorektalen Eingriffen [7] gefunden werden.

### Analyse akuter experimenteller Schmerz

Zu akutem experimentellen Schmerz wurden 7 randomisierte Cross-Over-Studien mit insgesamt *n* = 226 gesunden Proband*innen identifiziert. 6 der Studien [11, 22, 27, 33, 44, 75] konnten mit der Jadad-Skala bewertet werden und erreichten eine durchschnittliche Bewertung von 5,4 Punkten (Abb. 5). Die experimentellen Schmerzreize inkludierten Hitze bzw. Hitze und Druck (4 Studien [11, 19, 33, 75]), Neurometer (1 Studie [44]), Kälte und Druck (2 Studien [19, 27]), und säureinduzierte Hypersensibilität (1 Studie [22]). In 6 der 7 Studien [11, 22, 27, 33, 44, 75] konnten Unterschiede in der Schmerzwahrnehmung bzw. -intensität (geringer) und der Schmerzgrenze (höher) während bzw. nach Stimulation mit aVNS festgestellt werden, vgl. Tab. 2. Die vorliegenden Studien zeigen, dass bei experimentellem Schmerz bei manchen Patient*innen keine Veränderung in Bezug auf die Schmerzwahrnehmung (Non-Responder) oder sogar ein pro-nozizeptiver Effekt [44] erzielt wird. Dies zeigt sich in einer verstärkten Schmerzwahrnehmung und geringerer Schmerzgrenze während und direkt nach der Stimulation. Die Studienlage ergibt hier kein konsistentes Ergebnis.

### Studiendesigns

Von den ausgewerteten 37 Studien wurden 18 Studien mit Geräten zur pVNS (Nadelelektroden) [1, 7, 14, 15, 31, 35, 39–41, 45, 48, 62, 64–67, 71, 79] und 19 Studien mit Geräten zur tVNS (Oberflächenelektroden) [5, 11, 17, 19, 22, 24, 27, 30, 33, 43, 44, 46, 49, 53, 69, 70, 74, 75, 84] durchgeführt (vgl. Tab. 2). Die Stimulationselektroden wurden in vagal innervierten, aber zum Teil auch in nicht vagal innervierten Bereichen der Ohrmuschel platziert. Dies erfolgte abhängig vom verwendeten Gerät und den Erfahrungswerten der Studienautoren, wobei die Concha den am meisten verwendeten Stimulationsbereich darstellt. Die häufigste Kontrollgruppe war eine Sham-aVNS-Gruppe unter Verwendung eines inaktiven Geräts mit den Elektroden an der gleichen Position wie in der Interventionsgruppe (15 Studien [5, 7, 11, 31, 33, 35, 40, 41, 44, 45, 48, 65–67, 75]); gefolgt von einer aktiven Kontrolle mit Sham-aVNS an anderen nicht vagal innervierten Positionen und aktivem Gerät, wie z. B. am Ohrläppchen (7 Studien [19, 22, 33, 53, 69, 74, 84]); oder anderen Therapien als Kontrolle (pro- und retrospektiv, 5 Studien [1, 14, 15, 74, 79]). Eine Studie [70] verwendete eine aktive Kontrolle an den gleichen Punkten mit einem anderen Stimulationsmuster. In 1 Studie [43] wurde die aVNS mit physischem Training und in 1 Studie [53] synchron mit der Atmung durchgeführt.

### Stimulationsmuster

Es wurden die am Markt verfügbaren Geräte zur aVNS mit den in diesen Geräten entsprechend vorkonfigurierten Stimulationsparametern verwendet (vgl. Tab. 2). Die eingesetzten Geräte verwenden monophasische oder biphasische Rechteckimpulse mit einer Impulsbreite von 0,2–1 ms und Wiederholfrequenzen von 1–100 Hz. Die Strom- bzw. Spannungsamplitude war entweder konstant eingestellt oder wurde individuell an die Wahrnehmung der Patient*innen angepasst (von „subthreshold“ bis deutlich wahrnehmbar bzw. gerade noch tolerierbar). Die häufigste Konfiguration waren biphasische Rechteckimpulse mit 1 ms und 1 Hz sowie einer Amplitude, die eine deutliche, nicht schmerzhafte Wahrnehmung hervorgerufen hat.

### Verträglichkeit

In der Mehrzahl der Patient*innen bzw. Proband*innen wurden keine unerwünschten Ereignisse in Verbindung mit der aVNS beobachtet (vgl. Tab. 2). Die dokumentierten unerwünschten Ereignisse umfassten vorwiegend leichte Nebenwirkungen insbesondere lokal am Stimulationsort, wie Hautirritationen, Schmerzen und leichte Blutungen an den Einstichstellen von Nadelelektroden. Seltener wurde über Schwindel, Übelkeit, oder Müdigkeit berichtet. Ein Patient kollabierte hervorgerufen durch eine Nadelphobie.

Die Verträglichkeit des Geräts (sofern erhoben) wurde von einer bedeutenden Mehrheit der Patient*innen (> 75 %) als gut bis exzellent eingestuft.

## Diskussion

Chronische Schmerzen beeinträchtigen die Gesundheit und individuelle Lebensqualität von Patient*innen bedeutend [9]. Die Weiterentwicklung und Ergänzung effektiver Therapien für die Behandlung dieser Patient*innen wie auch Ansätze zur Vermeidung einer Chronifizierung von Schmerzen, z. B. nach einem operativen Eingriff, sind von hoher klinischer Relevanz. Die vorliegende systematische Übersichtsarbeit zeigt, dass die aVNS eine ergänzende, effektive nichtmedikamentöse Behandlung für Patient*innen mit spezifischen chronischen und akuten postoperativen Schmerzen ist.

Die Ergebnisse belegen eine konsistente schmerzreduzierende Wirkung sowie eine Verbesserung der Lebensqualität bei chronischen Schmerzpatient*innen in den Indikationen chronischer Rückenschmerz, Unterleibsschmerz und Migräne bei gleichzeitig sehr geringem Nebenwirkungsprofil (vgl. Tab. 2) auch bei Langzeitanwendung [49, 70, 71]. Wo erhoben, konnte in den analysierten Studien ein nachhaltiger therapeutischer Effekt erzielt werden, insbesondere in Bezug auf eine anhaltende signifikante Schmerzreduktion, einen geringeren Schmerzmittelbedarf und ein besseres psychisches Wohlbefinden (vgl. Tab. 2). Diese nachhaltige Wirkung hielt über Wochen bis Monate nach Stimulationsende an (Follow-up zwischen 2 Wochen und max. 12 Monaten) [5, 40, 49, 62, 64–66, 71]. Eine solche lang anhaltende Wirkung der aVNS wurde bereits früher in anderen Indikationen gezeigt, so z. B. bei Epilepsie oder Depression [34, 54, 82]. Als mögliche Wirkmechanismen werden die Aktivierung neuroplastischer Effekte in den in die Schmerzverarbeitung involvierten zentralen Hirn- und Rückenmarkstrukturen (Wirkung auf zentrale Sensitivierung) sowie eine antiinflammatorische Wirkung z. B. auf neuroinflammatorische Prozesse angenommen [23, 37].

Die Verträglichkeit der Behandlung kann auf Basis der vorliegenden Literaturanalyse als sehr gut bewertet werden. Wechselwirkungen mit medikamentösen Begleittherapien konnten in den untersuchten Studien nicht festgestellt werden. Das Nebenwirkungsprofil der aVNS wurde bereits in anderen Studien und Indikationen als sehr gut bewertet [23, 60, 82]. Eine retrospektive Analyse von Roberts et al. [61] dokumentierte bei 1207 Anwendungen von perkutaner aVNS nur 24 (1,98 %) unerwünschte Ereignisse, bestehend aus leichten Blutungen an der Einstichstelle der Nadelelektroden, lokaler Dermatitis und Schmerz an der Einstichstelle. Systemische Nebenwirkungen oder Infektionen wurden nicht beobachtet. Auch Untersuchungen zu möglichen kardiovaskulären Nebenwirkungen der aVNS ergaben kein erhöhtes Risiko [42]. In Anwenderstudien wurde zudem die Zufriedenheit der Patient*innen mit perkutaner aVNS bei mehrwöchiger Anwendung erhoben. 80 % der Patient*innen beschrieben dabei die Behandlung mittels aVNS als sehr zufriedenstellend in Bezug auf ihre subjektive Wahrnehmung der Lebensqualität [36].

Eine solche nachhaltige therapeutische Verbesserung und gute Verträglichkeit ist nicht nur von individueller Bedeutung, sondern auch gesamtgesellschaftlich und ökonomisch höchst relevant aufgrund der enormen sozioökonomischen Kosten chronischer Schmerzen [47]. Für die nichtinvasive zervikale Vagusnervstimulation konnten entsprechend Kostenersparnisse bei gleichzeitig größerem Gesundheitsvorteil bei chronischem Clusterkopfschmerz und episodischer Migräne bereits gezeigt werden [50–52]. Zukünftige Studien mit aVNS sollten im Hinblick auf die klinische und mögliche sozioökonomische Bedeutung auch diesen Aspekt, vor allem unter Berücksichtigung standardisierter Behandlungsparameter, längerer Beobachtungzeiträume sowie der Erfassung der Lebensqualität und Arbeitsfähigkeit betroffener Patient*innen, näher beleuchten [23].

Die Mechanismen im chronischen Schmerzgeschehen unterscheiden sich grundlegend von jenen des akuten Schmerzes [23, 37]. In Bezug auf die Wirkung der aVNS bei akuten Schmerzen zeigen sich inkonsistente Ergebnisse. Bei einigen Studien konnte keine Wirkung auf das postoperative Schmerzgeschehen oder den Medikamentenbedarf gezeigt werden [31, 48]. Eine Subgruppenanalyse bei Blank et al. [7] zeigt keinen Effekt in der gesamten Kohorte, jedoch einen signifikanten Effekt in der Subgruppenanalyse bei offenen kolorektalen Eingriffen. Eine Wirkung wurde eher bei schwereren Eingriffen mit stärkerem Trauma und Entzündungsgeschehen beobachtet im Vergleich zu minimal-invasiven Eingriffen. Die Ergebnisse der Studien zu akutem experimentellem Schmerz in gesunden Proband*innen lieferten ebenfalls inkonsistente Ergebnisse. Detailliertere Betrachtungen hierzu finden sich in folgenden Arbeiten: [23, 76, 82].

### Limitationen

Trotz einer größeren Zahl gut durchgeführter Studien zur Wirkung der aVNS bei chronischen (9 RCTs mit *n* = 255 aVNS-stimulierten Patient*innen) und akuten (7 RCTs zu postoperativem Schmerz mit *n* = 181 aVNS-stimulierte Patient*innen) Schmerzen, sind die Studien untereinander oft nur bedingt direkt vergleichbar. In den vorliegenden Studien sind Unterschiede in der verwendeten Kontrollgruppe, der Lokalisation der Stimulationselektroden, der Stimulationsparameter und der Behandlungsdauer immanent, vgl. Tab. 2. Ein abschließender Konsens bzgl. der Auswirkungen dieser Unterschiede auf die Wirksamkeit der aVNS in vorliegenden Indikationen besteht nicht. Ebenso gibt es noch keine allgemeingültigen Leitlinien oder Anwendungsanleitungen zu indikationsspezifischen Stimulationsparametern und Behandlungsdauern [23, 76, 77, 82]. Die klinische Wirksamkeit kann durch diese Parameter entscheidend beeinflusst werden, weshalb dieser Aspekt von hoher klinischer Relevanz ist.

Aufgrund der dichten Innervierung der Ohrmuschel nicht nur durch den Vagusnerv, sondern auch durch den aurikulotemporalen Nerv (Ast des Trigeminusnervs), den großen aurikulären Nerv und den Nervus occipitalis minor, kann bei den hier zusammengefassten und analysierten Studien auch von einer Ko-Stimulation nichtvagaler Fasern ausgegangen werden [25, 59, 83]. Dies ist speziell bei der Wahl der Kontrollgruppe von Relevanz. In einer Sham-Kontrolle mittels Stimulation z. B. am Ohrläppchen kann nicht davon ausgegangen werden, dass diese Stimulation wirkungslos ist. Durch die Stimulation des großen aurikulären Nervs können Wirkungen auftreten, die noch nicht näher erforscht sind. So gibt es Hinweise, dass durch Stimulation des großen aurikulären Nervs eine therapeutische Wirkung bei Migräne oder Clusterkopfschmerz erzielt werden kann [20]. Auch in fMRI-Studien konnte eine entsprechende Modulation spezifischer Hirnregionen bei Ohrläppchenstimulation gezeigt werden [25]. Eine hohe lokale Spezifität bei gleichzeitiger effizienter und individualisierter Stimulation scheint essenziell für eine erfolgreiche Therapie mit aVNS. Das ist durch die Verwendung von Nadelelektroden, wie in der perkutanen aVNS, besser erfüllbar verglichen mit Oberflächenelektroden in der transkutanen aVNS.

Um zukünftig eine bessere und vergleichbare Evidenzlage zu ermöglichen, hat ein rezenter Konsensus-Review von Farmer et al. [23] die Bedeutung von minimalen Reporting-Kriterien in Studien zu aVNS hervorgehoben. Ein aktueller systematischer Review von Wang et al. [78] evaluiert auch die Bedeutung der noch sehr unterschiedlichen Nomenklatur bei Bezeichnung der aVNS (u. a. aurikuläre transkutane elektrische Nervenstimulation, periphere elektrische Nervenfeldstimulation, elektrische Punktualstimulation oder aurikuläre Neurostimulation), die ebenfalls einer Standardisierung bedarf, um eine konsistente klinische Bewertung möglich zu machen.

Zusätzlich ist die Erforschung und Festlegung von Kriterien, die über den individuellen Behandlungserfolg von Patient*innen entscheiden, von hoher Relevanz [37]. So gibt es Hinweise, dass es Unterschiede bei der Wirksamkeit der aVNS bei neuropathischen und somatischen Schmerzen gibt [65], ebenso bezüglich des Einflusses der affektiven Komponenten [26]. Dies wird zum Teil in den vorliegenden Studien noch zu wenig beleuchtet. Die Einbindung verschiedener physiologischer Messwerte individueller Patient*innen vor Beginn der Therapie könnte dabei helfen. Es gibt Versuche, z. B. auf Basis der Herzratenvariabilität bzw. des autonomen Status eines/r Patienten/in die Wahrscheinlichkeit des therapeutischen Ansprechens dieses/r Patienten/in vorherzusagen und damit die Patientenauswahl im Hinblick einer positiven Therapieprognose zu vereinfachen [10, 37]. Ein solcher Ansatz wird auch aktuell in Studien bei chronischen Kreuzschmerzpatient*innen untersucht (clinicaltrials.gov NCT04753528) Entsprechende Leitfäden zur Patientenselektion werden essenziell für den klinischen Einsatz der Methode bei chronischen und akuten Schmerzen sein.

## Fazit für die Praxis

Die Methode der aurikulären Vagusnervstimulation ist einfach in der Anwendung und hat ein geringes Nebenwirkungsprofil. Der Einsatz aurikulärer Vagusnervstimulation kann eine effektive Ergänzung zur multimodalen Schmerztherapie bei chronischen Rückenschmerzen, Unterleibsschmerzen, und Migräne darstellen. Da die Ergebnisse bei akuten experimentellen und postoperativen Schmerzen nicht eindeutig sind, müssen weitere Studien folgen, um herauszufinden, bei welchen pathophysiologischen Schmerzursachen bzw. bei welchen Eingriffen die aurikuläre Vagusnervstimulation besonders effektiv sein kann.

### Supplementary Information

Tab. 2 Zusammenfassung und Analyse der Studien zur Anwendung der aVNS bei chronischen Schmerzen, akuten postoperativen Schmerzen und akuten experimentellen Schmerzen – Langversion.
