# Supplementary material for: Clinical efficacy of auricular vagus nerve stimulation in the treatment of chronic and acute pain: A systematic review
Source: Schmerz. 2023 Jan 2;38(4):267–78. [Article in German] doi: 10.1007/s00482-022-00686-2 (PMC11271441; doi:10.1007/s00482-022-00686-2)
Supplement: Supplementary file 1 — Tab. 2 Zusammenfassung und Analyse der Studien zur Anwendung der aVNS bei chronischen Schmerzen, akuten postoperativen Schmerzen und akuten experimentellen Schmerzen – Langversion. [file 482_2022_686_MOESM1_ESM.pdf]

Tab. 2 Zusammenfassung und Analyse der Studien zur Anwendung der aVNS bei chronischen Schmerzen, akuten postoperativen Schmerzen und akuten experimentellen Schmerzen - Langversion. [a]

**Studie**	**Indikation**	**Methode [b]**	**Probandenanzahl (Dropouts)**	**Stimulations-parameter**	**Behandlungs-dauer**	**Primäres Ergebnis [c]**	**Sekundäres Ergebnis**	**Unerwünschte Ereignisse**
**chronischer Schmerz**								
Sator-Katzenschlager et al. 2003 [66]	chronisches Zervikalsyndrom	pVNS: 2 x Antitragus, <u>Fossa triangularis</u>; rechts oder links vs. Sham-pVNS: inaktives Gerät	*n=23*<br><br>pVNS=10 (1)<br>Sham-pVNS=11 (1)	P-Stim (Biegler)<br><br>Rechteck, monophasisch, alternierende Polarität, 1 Hz, 1 ms, 2 mA (3.8V) konstant, 3h ON/3h OFF	48h/W für 6 Wochen<br><br>4 Woche Follow-Up	signifikante Reduktion ***VAS*** Schmerzskala (pVNS vs. Sham, p<0.05)<br><br>signifikante Reduktion blieb über ***4 Wochen FU*** (p<0.05) erhalten	signifikante Verbesserungen bei ***psychischem Wohlbefinden***, ***Aktivität***, und ***Schlaf*** (pVNS vs. Sham, p<0.05)<br><br>**Schmerzmittelbedarf** signifikant niedriger pVNS vs. Sham	keine behandlungs-relevanten UEs
Sator-Katzenschlager et al. 2004 [65]	chronischer Kreuzschmerz	pVNS: <u>Antihelix</u>, <u>Fossa triangularis</u>, Antitragus, rechts oder links vs. Sham-pVNS: inaktives Gerät	*n=61*<br><br>pVNS=29 (2)<br>Sham-pVNS=26 (4)	P-Stim (Biegler)<br><br>Rechteck, monophasisch, alternierende Polarität, 1 Hz, 1 ms, 2 mA (3.8V) konstant, 3h ON/3h OFF	48h/W für 6 Wochen<br><br>3 Monate Follow-Up	signifikante Reduktion ***VAS*** Skala (p-VNS vs. Sham, p<0.05)<br><br>signifikante Reduktion blieb über ***3 Monate FU*** (p<0.05) erhalten	signifikante Verbesserungen bei ***psychischem Wohlbefinden***, ***Aktivität***, und ***Schlaf*** (pVNS vs. Sham (p<0.05)<br><br>**Schmerzmittelbedarf** signifikant niedriger pVNS vs. Sham	keine behandlungs-relevanten UEs

Kong et al. 2009 [39]	chronischer Schmerz (Spondylose, Migräne)	pVNS: 3 aus Fossa triangularis, Crus superius antihelicis, Antitragus, Crus inferius antihelicis, Conchae, rechts oder links	*n=9*<br><br>(Migräne n=2, Spondylose n=7)<br>pVNS=6 (3)	P-Stim (Biegler)<br><br>Rechteck, monophasisch, alternierende Polarität, 1 Hz, 1 ms, 3.8V (2 mA) konstant, 3h ON/3h OFF	4 Tage/W für 2 Wochen<br><br>2 Wochen Follow-Up	VAS-Reduktion von 7 (Behandlungsstart) auf 4 (Woche 1), 2 (Woche 2), bzw. 3 (Woche 4) (jeweils Wochen-Mittelwerte)<br><br>Verbesserungen blieben für bis zu 2 Wochen nachhaltig bestehen	k.A.	lokaler Schmerz an Stimulationspunkt (n=3)<br>Übelkeit (n=2)<br>Schwindel (n=1)<br>lokaler Juckreiz (n=2)
Napadow et al. 2012 [53]	chronischer Beckenschmerz (CPP)/Endometriose	tVNS (Atemsynchron): Cymba Conchae, zw. Antihelix und Cavum Conchae, links<br>vs.<br>Sham-tVNS (nicht Atemsynchron): Ohrläppchen, links	*n=18*<br><br>tVNS=15 (3)	Cefar Acus II (Cefar Medical)<br><br>Rechteck, 30 Hz, 0.45 ms, Amplitude individuell auf deutliche Wahrnehmbarkeit angepasst, Stimulation synchronisiert/nicht synchronisiert mit Ausatmung	30 Minuten	Trend zu reduzierter evozierter Schmerzintensität unter tVNS (Atemsynchron) vs. Sham-tVNS (nicht Atemsynchron)	keine signifikanten Veränderungen oder Unterschiede auf NRS-Schmerskala<br><br>tVNS (Atemsynchron) bewirkte signifikant stärkere ***Abnahme*** der ***Angst***-Werte vs. Ausgangswert ($p<0.01$) und vs. Sham-tVNS (nicht Atemsynchron, $p<0.05$)	k.A.
Straube et al. 2015 [70]	chronische Migräne	tVNS (25 Hz): Cymba Conchae, links<br>vs.<br>tVNS (1 Hz): Cymba Conchae, links	*n=46*<br><br>tVNS (25 Hz)=22 (2)<br>tVNS (1 Hz)=17 (5)	NEMOS (Cerbomed)<br><br>Rechteck, biphasisch, 25 Hz bzw. 1 Hz (Kontrolle), 0.25 ms, Amplitude individuell auf max. tolerierbare Wahrnehmbarkeit, 30s ON/30s OFF	4h/T für 3 Monate	signifikant größere Reduktion an ***Kopfschmerztagen/ 28 Tage*** tVNS (1Hz) vs. tVNS (25Hz) ($p=0.035$)	Prozentsatz Responder 29.4% vs. 13.6% (1Hz vs. 25 Hz, Per Protokoll Analyse)<br><br>keine signifikante Veränderung der Kopfschmerzintensität ggb. Ausgangswert und 1Hz vs. 25 Hz<br><br>**Kopfschmerzmedikationstage**, **MIDAS** und **HIT-6** in beiden	milder bis moderater Schmerz, Parästhesie, oder Pruritus während oder nach der Stimulation; Erythem, Ulzeration oder Schorf<br><br>Behandlungsrelevante UEs:

							Gruppen signifikant reduziert ggb. Ausgangswert, keine Unterschiede zwischen den Gruppen	n=112, davon n=76 tVNS-induziert (25 Hz); n=67, davon n=39 tVNS-induziert (1 Hz)
Sacco et al. 2016 [62]	Chemotherapie-induzierte periphere Neuropathie	pVNS: <u>Fossa Triangularis</u>, <u>Tragus</u>, <u>Antihelix</u> und/oder <u>Conchae</u>	*n=58*<br><br>pVNS=58 (0)<br><br>primärer Endpunkt=18 sekundärer Endpunkt=32 (8)	P-Stim (Biegler)<br><br>Rechteck, monophasisch, alternierende Polarität, 1 Hz, 1 ms, 3.8V (2 mA) konstant, 3h ON/3h OFF	5 Tage/W für durchschnittlich 5 Wochen	signifikante Verbesserung ggb. Ausgangswert um 5 Punkte (Median) auf ***NRS***-Skala (p<0.001)	59% deutliche Schmerzreduktion, 12.4% bemerkbare Schmerz- und Taubheitsgefühlreduktion, 29% Non-Responder<br><br>nachhaltiger Therapieeffekt bei allen Respondern<br><br>funktionelle Verbesserungen insbesondere bei ADL, Gang und Balance<br><br>Reduktion/Absetzen der Schmerzmedikation (n=6)	Stimulation nicht tolerierbar (n=1)

Kovacic et al. 2017 [40]	chronischer Unterleibschmerz<br><br>Jugendliche 11-18 Jahre	pVNS: Ohrläppchen, Fossa triangularis/Crus superius antihelicis, Conchae (posterior), temporale Region (nicht in der Ohrmuschel) vs. Sham-pVNS: inaktives Gerät	*n=115*<br><br>pVNS=57 (3/FU7) Sham-pVNS=47 (8/FU4)	Neuro-Stim (Innovative Health Solutions)<br><br>Rechteck, 1/10 Hz alternierend alle 2s, 1 ms, 3.2 V konstant, 2h ON /2h OFF	5 Tage/W für 4 Wochen<br><br>Follow-Up 8-12 Wochen<br><br>Fragebogen 6-12 Monate nach Therapieende	Nach 3 Wochen: Reduktion schwerer Schmerzen, ***PFSD*** (pVNS vs. Sham, p<0.0001)<br><br>Behandlungseffekt blieb bis inkl. ***FU*** bestehen	FDI-Skala: keine Veränderung bei Sham ggb. Ausgangswert (moderate Einschränkungen, beide Zeitpunkte)<br><br>***FDI-Skala***: signifikante Verbesserung bei pVNS ggb. Ausgangswert, moderate Einschränkungen vs. minimale Einschränkungen, Verbesserung um durchschnittlich 36%<br><br>keine Veränderungen im STAI-C in beiden Gruppen	Ohr-Diskomfort (pVNS/Sham n=3/3)<br>Allergie gegen Klebstoff (pVNS/Sham n=3/2)<br>Synkope durch Nadelphobie (pVNS/Sham n=0/1)
Grolaux et al. 2019 [30]	IBS-Schmerz und chronischer Schmerz	tVNS: Conchae, links	*n=6*<br><br>tVNS=6 [IBS=3, chron. Schmerz=3] (0)	TU7000 (Digital TENS, RMP)<br><br>Rechteck, biphasisch, (chronischer Schmerz: 5 Hz, 0.2 ms) / (IBS: 3 Hz, 0.25 ms), Amplitude individuell auf Wahrnehmbarkeit (~0.8 mA)	2 x 30 Min/W für 4 Wochen<br><br>Follow-Up nach 2 Monaten	keine signifikanten Änderungen im BPI-Fragebogen (Kurzform)<br><br>klinische Verbesserung (IBS-SSS) bei der IBS-Gruppe beobachtbar	k.A.	Nackenschmerz (n=6)<br>Juckreiz an Stimulationsstelle (n=2)<br>Übelkeit (n=2)<br>Kopfschmerz (n=3)

Krasaelap et al. 2019 [41]	IBD-Schmerz	pVNS: Fossa triangularis, Cymba Conchae, Ohrläppchen (Referenz)<br>vs.<br>Sham-pVNS: inaktives Gerät	*n=50*<br><br>pVNS=27 (0)<br>Sham-pVNS=23 (0)	Neuro-Stim (Innovative Health Solutions)<br><br>Rechteck, 1/10 Hz alternierend alle 2s, 1 ms, 3.2V, 2h ON/2h OFF	5 Tage/W für 4 Wochen	59% der Patient*innen in der aktiven Therapiegruppe vs. 26% in der Sham-Gruppe erreichten eine Reduktion ≥30% bei ***schweren abdominalen Schmerzen*** (Ausgangswert vs. 3 Wochen Therapie, p=0.024)	nach 3 Wochen ***zusammengesetzte Schmerzskala*** Reduktion pVNS vs. Sham-pVNS (p=0.026)<br><br>nach 3 Wochen ***üblicher Schmerz*** Reduktion pVNS vs. Sham-pVNS (p=0.029)<br><br>Verbesserung bei ***allgemeinen/globalen Symptomen*** pVNS vs. Sham-pVNS (p<0.001)	Allergie gegen Klebestoff (n=1)
Mion et al. 2020 [49]	IBS-Schmerz	tVNS: Conchae, links	*n=12*<br><br>tVNS=9 (3)	Urostim 2 (Schwa-medico)<br><br>Rechteck, 30 Hz, 0.25 ms, 0.5-20 mA (individuell unter Wahrnehmungsschwelle)	3h/Tag für 5 T/Woche für 6 Monate	signifikante Verringerung beim ***IBS-SSS*** nach 3 (p = 0.0084) und 6 Monaten (p = 0.0209) ggb. Ausgangswert<br><br>klinisch signifikantes Ansprechen (min. 30% Reduktion im IBS-SSS) bei 5 Patient*innen nach 3 Monaten (2 IBS-D, 3 IBS-C), bei 4 nach 6 Monaten (2 IBS-C, 2 IBS-D)	***Schwere und Frequenz des abdominalen Schmerzes*** signifikant geringer nach 1 Monat tVNS und bis 6 Monate (p<0.03)<br><br>keine signifikanten Veränderungen bei QoL - SF-36, psychologischem Wohlbefinden - PANAS, STAI, CES-DS, PSS, WCC; inflammatorischen Zytokine und fäkalem Calprotectin<br><br>Begleitmedikation konnte reduziert werden	keine

Kutlu et al. 2020 [43]	Fibromyalgie	physisches Training + tVNS: Tragus und Conchae, bilateral<br>vs.<br>physisches Training	*n=60*<br><br>tVNS + Training=27 (3)<br>Training=25 (5)	TENS-Gerät<br><br>biphasisch, asymmetrisch, 10 Hz, 0.5 ms, Amplitude individuell (Wahrnehmungsschwelle)	30 Min/T, 5 Tage/W für 4 Wochen	beide Gruppen zeigten statistisch signifikante Verbesserungen bei Schmerz (***VAS***), Depression (***Beck Depression Scale***), ***Angst***, ***Funktionalität***, und im ***Life Quality Score*** (SF-36)	k.A.	keine
Shi et al. 2021 [69]	IBS-C, chronischer abdominaler Schmerz	tVNS: Cymba Conchae, bilateral<br>vs.<br>Sham (nicht aurikuläre Stimulation): Ellbogen, bilateral	*n=42*<br><br>tVNS=21 (0)<br>Sham=19 (2)	SNM-FDC01 (Ningbo Maida Medical Device)<br><br>Rechteck, 25 Hz, 0.5 ms, 0–2 mA (maximal tolerierbare Amplitude), 2s ON/3s OFF	2x30 Min/T für 4 Wochen	tVNS erhöhte signifikant ***CSBMs/Woche*** (p=0.001) und reduzierte signifikant den ***VAS***-Wert von abdominalen Schmerzen (p=0.001) vs. Sham	tVNS vs. Sham: verbesserte ***Lebensqualität*** (p=0.020), verringerter ***IBS-SSS*** (p=0.001)<br><br>verbesserter ***rektoanaler inhibitorischer Reflex*** (p=0.014), verbesserte ***rektale Empfindlichkeit*** (p<0.04); niedrigere Level an proinflammatorischen Zytokinen und Serotonin<br><br>verstärkte ***vagale Aktivität*** (EKG, p=0.040)	keine

Széles et al. 2021 [71]	Behandlungs-resistenter chronischer Rückenschmerz	pVNS: Cymba Conchae, Conchae, Crus antihelicis, links und rechts alternierend	*n=148*<br><br>pVNS=148 (0)	P-Stim (Biegler)<br><br>Rechteck, monophasisch, alternierende Polarität, 1 Hz, 1 ms, 3.8V (2 mA) konstant, 3h ON/3h OFF	4 Tage/W für mind. 3 Wochen und bis zu 3 Monate	≥30% Reduktion der durchschnittlichen ***NRS-*** Schmerzintensität (ITT): 51.4% (Woche 1), 70.3% (Woche 3), 72.3% (Woche 6), 75.0% (3 Monate)<br><br>ITT-Population: 32.4% erreichten ≥ 50% Verbesserung (Definition Responder) der durchschnittlichen Schmerzintensität (Woche 1), 49.3% (Woche 3), 58.8% (Woche 6)	subjektives Wohlbefinden: Verbesserung um durchschnittlich 1.89 Punkte (±1.66; n=54)<br><br>49.3% reduzierten oder beendeten ihre Schmerzmedikation (n=67)<br><br>verbesserte Mobilität (n=20), verbesserte Schlafqualität (n=3), Reduktion psychischer Anspannung (n=1)	unbeabsichtigtes Lösen des Gerätes (n=20) unbeabsichtigte Unterbrechung der Stimulation (n=15) Hautirritationen (n=4) Schwindel/Kopfweh/Schmerz an Stimulationsstelle (n=1)
Aranow et al. 2021 [5]	SLE und muskuloskelettaler Schmerz	tVNS: Conchae (anterior und posterior) vs. Sham-tVNS: Ohrläppchen, inaktives Gerät	*n=18*<br><br>tVNS=12 (0) Sham-tVNS=6 (0)	TENS 7000 (Roscoe)<br><br>Rechteck, 30 Hz, 0.3 ms, maximal tolerierbare Amplitude	5 Min/T für 4 Tage	tVNS bewirkte signifikante Reduktion bei ***Schmerz*** und ***Müdigkeit*** vs. Sham-tVNS	PtGA-Werte, Anzahl geschwollener/betroffener Gelenke und PGA-Werte tVNS vs. Sham-tVNS verbessert<br><br>Effekte blieben bis Tag 12 bestehen<br><br>Plasmakonzentration von ***Substanz P*** signifikant reduziert tVNS ggb. Ausgangswert	keine
Woodbury et al. 2021 [79]	Fibromyalgie	pVNS: Conchae (posterior), Crus helicis, Crus superius antihelicis, Ohrläppchen, rechts oder links vs.	*n=21*<br><br>pVNS=12 (0) Kontrolle=9 (0)	NSS (EAD MFS, BRIDGE; Innovative Health Solution)<br><br>Rechteck, 1/10 Hz alternierend alle 2s, 3.2V, 1 ms, 2h ON/2h OFF	5 Tage/W für 4 Wochen<br><br>12 Wochen Follow-Up	nicht-signifikante Verbesserung auf der Schmerzskala pVNS vs. Kontrolle<br><br>signifikante Verbesserungen bei ***Schlaf, Aktivität,***	im fMRI feststellbare verstärkte Gehirnkonnektivität (Cerebellum, in exekutiven Kontroll-Netzwerken) in der pVNS-Gruppe nach	Leichte Irritationen an Stimulationsstelle (n=2)

		Standardtherapie				***Stimmung*** in Woche 6 in pVNS-Gruppe vs. Kontrolle	Behandlung vs. Kontrolle	
Marsal et al. 2021 [46]	Rheumatoide Arthritis	tVNS: Cymba Conchae	*n=30*<br><br>tVNS=27 (3)	Nẽsos<br><br>Rechteck, 20 kHz, Amplitude individuelle Wahrnehmungsschwelle (~3 mA)	30 Min/T für 12 Wochen<br><br>Follow-Up: W1, W2, W4, W8, W12 (Therapieverlauf)	nach Woche 12 signifikante Änderung im ***DAS28-CRP*** (p<0.0001) ggb. Ausgangswert	Reduktion ***HAQ-DI*** (p<0.0001) ggb. Ausgangswert	Hautabschürfung (n=1);<br><br>klassifiziert als nicht Behandlungs-relevant:<br>Sturz (n=2)<br>Mucus im Hals (n=1)
Zhang et al. 2021 [84]	Migräne ohne Aura	tVNS: Cymba Conchae, links vs.<br>Sham-tVNS: Cauda helicis, links	*n=63*<br><br>tVNS=33 (1)<br>Sham-tVNS=26 (3)	SDZII (Huatuo)<br><br>Rechteck, 1 Hz, 0.2 ms, maximal tolerierbare Amplitude	30 Min/Einheit, 12 Einheiten in 4 Wochen	signifikante Reduktion bei ***Anzahl der Migränetage*** (p<0.05), ***Schmerzintensität*** (p<0.01), und ***Dauer der Migräneattacken*** (p<0.05) tVNS vs. Sham-tVNS nach 4 Wochen Behandlung<br><br>signifikante Reduktion der ***Migränetage*** in der tVNS-Gruppe ggb. Ausgangswert (p<0.001)	funktionelle Konnektivitätsanalyse im fMRI: tVNS erhöht die Konnektivität zwischen motorisch-verknüpften Thalamus-Subregionen und dem anterior cingulate Cortex/medialen präfrontalen Cortex, während die Konnektivität zwischen occipital-verknüpfter Thalamus-Subregion und postzentralem Gyrus/Präcuneus herabgesetzt wird	k.A.

Feng et al. 2022 [24]	Migräne ohne Aura	tVNS: Cymba Conchae, links<br><br>fMRI-Kontrollgruppe mit gesunden Probanden	*n=60*<br><br>tVNS=60 (0)<br>Kontrolle=60(0)	SDZII (Huatuo)<br><br>Rechteck, 1 Hz, 0.2 ms, maximal tolerierbare Amplitude (1.5-5 mA)	30 Min/Einheit, 12 Einheiten über 4 Wochen	signifikante Verbesserung ***VAS, Attackenanzahl***, ***Dauer***, ***MSQ***, ***SDS***, ***SAS*** (p<0.05) ggb. Ausgangswert<br><br>keine signifikanten Veränderungen bei Notfallmedikation (Ibuprofen)	im fMRI: Veränderungen im rechten PoCG nach tVNS negativ assoziiert mit VAS-Reduktion, im bilateralen Präcuneus positiv assoziiert mit Dauer von Migräneattacken	k.A.
Courties et al. 2022 [17]	Osteoarthritis/Handschmerz	tVNS: Cymba Conchae, links	*n=20*<br><br>tVNS=18 (2)	TENSeco 2 (Schwa-Medico)<br><br>biphasisch, asymmetrisch, balanciert, 25 Hz, 0.05 ms; Amplitude bis 15 mA bzw. maximal tolerierbar	1h/T für 4 Wochen	signifikante Reduktion des ***VAS*** ggb. Ausgangswert (p=0.001)	signifikante Reduktion bei ***FIHOA*** (p = 0.01)<br><br>12 Patient*innen hatten weniger schmerzende Gelenke, 15 weniger geschwollene Gelenke<br><br>keine signifikante Veränderung bei Paracetamol Bedarf	Diskomfort am Ohr (n=1)

Santucci et al. 2022 [64]	chronischer funktioneller Unterleibschmerz<br><br>Jugendliche 11-18 Jahre	pVNS: Ohrläppchen, Fossa triangularis/Crus superioris antihelicis, Conchae (posterior), temporale Region (nicht in der Ohrmuschel)	*n=20*<br><br>pVNS=19 (1)	IB-Stim (Innovative Health Solutions)<br><br>Rechteck, 1/10 Hz alternierend alle 2s, 1 ms, 3.2V konstant, 2h ON/35 Min OFF	5 Tage/W für 4 Wochen	WL-SPT-induzierte ***VAS*** (p=0.004) und ***Übelkeit*** (0.02) geringer ggb. Ausgangswert;<br><br>***Ruhe-VAS*** (p=0.03), ***abdominaler Schmerz*** (p<0.0001), ***Schmerz Katastrophierung*** (p=0.0004), ***somatische Beschwerden*** (p=0.01), ***funktionelle Einschränkungen*** (p=0.04), und ***Ängstlichkeit*** (p=0.02) zeigten signifikante Verbesserungen ggb. Ausgangswert<br><br>einige Effekte langanhaltend für 6-12 Monate nach Therapie	***Schlaf*** (p<0.05) und ***Einschlaflatenz*** (p=0.03) verbesserten sich signifikant	k.A.

akuter postoperativer Schmerz								
Sator-Katzenschlager et al. 2006 [67]	perioperativer Schmerz (Oozyten-Aspiration)	pVNS: 2 x <u>Fossa triangularis</u>, Antitragus, links oder rechts<br>vs.<br>Sham-pVNS 1: inaktives Gerät<br>vs.<br>Sham-pVNS 2: nadellose Elektroden, inaktives Gerät	*n=94*<br><br>pVNS=32 (0)<br>Sham-pVNS 1=32 (0)<br>Sham-VNS 2=29 (1)	P-Stim (Biegler)<br><br>Rechteck, monophasisch, alternierende Polarität, 1 Hz, 1 ms, 3.8V (2 mA) konstant, 3h ON/3h OFF	Start 30 Min vor Eingriff bis 1h nach Eingriff-Ende	Schmerzlinderung (***VAS***) während bzw. nach Eingriff in pVNS-Gruppe signifikant größer im Vergleich zu beiden Sham-Gruppen (p<0.001)	subjektives ***Wohlbefinden*** in pVNS-Gruppe signifikant besser während und nach Eingriff (p<0.005)<br><br>***Opioidkonsum*** (Remifentanil) signifikant niedriger (p<0.001) in pVNS-Gruppe<br><br>Patient*innen in ***Sham-pVNS 2*** (ohne Nadelelektroden) waren signifikant ***müder*** (p<0.001)<br><br>Übelkeit in allen Gruppen vergleichbar	keine behandlungsrel evanten UEs
Likar et al. 2007 [45]	postoperativer Schmerz (laparoskopische Nephrektomie)	pVNS: <u>Cymba Conchae</u>, <u>Fossa triangularis</u>, Antitragus<br>vs.<br>Sham-pVNS: inaktives Gerät	*n=44*<br><br>pVNS=21 (1)<br>Sham-pVNS=20 (2)	P-Stim (modifiziert; Biegler)<br><br>Rechteck, monophasisch, alternierende Polarität, 1 Hz, 20 ms, Amplitude unterschwellig, 3h ON/3h OFF	Start 30 Min vor Eingriff, 96h gesamt	pVNS vs. Sham-pVNS: bessere ***Ruhe-VAS*** und ***Belastungs-VAS-Werte***, insbesondere in der 1. Stunde nach Eingriff (p<0.05)	postoperative ***Einnahme von Morphinhydrochlorid*** signifikant geringer pVNS vs. Sham-pVNS (p<0.05)<br><br>erster ***Analgetikabedarf signifikant später*** pVNS vs. Sham-pVNS (p<0.05)	Übelkeit und Erbrechen (pVNS/Sham n=5/1)

Michalek-Sauberer et al. 2007 [48]	postoperativer Schmerz (Backenzahn-Extraktion)	pVNS: Ohrläppchen, Fossa triangularis, Cavum Conchae, links oder rechts<br>vs.<br>vs.<br>Sham-pVNS 1: inaktives Gerät<br>vs.<br>Sham-pVNS 2: nadellose Elektroden, inaktives Gerät	*n=128*<br><br>pVNS=48 (15)<br>Sham-pVNS 1=26 (8)<br>Sham-VNS 2=26 (5)	P-Stim (modifiziert; Biegler)<br><br>Rechteck, monophasisch, alternierende Polarität, 2/100 Hz alternierend alle 3s, 1 ms, 3.8V (2 mA) konstant, 3h ON/3h OFF	Start 30 Min vor Eingriff, 48 Stunden	moderate bis schwere Schmerzdauer nicht signifikant unterschiedlich in den Gruppen<br><br>keine signifikanten Unterschiede in der wahrgenommenen Schmerzintensität	keine Unterschiede bei der Einnahme analgetischer Medikamente (Paracetamol, Mefenaminsäure)	Synkope (pVNS/Sham n=1/0)<br>Ohrenschmerz durch Stimulation (pVNS/Sham n=7/2)<br>Müdigkeit (pVNS/Sham n=10/3)
Kager et al. 2009 [35]	postoperativer Schmerz (Tonsillektomie)	pVNS: Fossa triangularis, Antitragus, Tuberculum auriculare<br>vs.<br>Sham-pVNS: inaktives Gerät	*n=33*<br><br>pVNS=16 (0)<br>Sham-pVNS=17 (0)	P-Stim (modifiziert; Biegler)<br><br>Rechteck, monophasisch, alternierende Polarität, 1 Hz, 20 ms, Amplitude unterschwellig, 3h ON/3h OFF	Start 30 Min vor Eingriff, 96h Stunden	***VAS*** signifikant niedriger pVNS vs. Sham-pVNS 9 (p=0.044), 12 (p=0.022) und 24h (p=0.045) postoperativ	Sham-pVNS hatte einen höheren Analgetikabedarf ggb. pVNS (nicht signifikant)	Übelkeit und Erbrechen (n=6)
Holzer et al. 2011 [31]	postoperativer Schmerz (gynäkologische Eingriffe)	pVNS: Fossa triangularis, Antitragus, 1 individueller Punkt<br>vs.<br>Sham-pVNS: nadellose Elektroden, inaktives Gerät	*n=40*<br><br>pVNS=20 (0)<br>Sham-VNS=20 (0)	P-Stim (Biegler)<br><br>Rechteck, monophasisch, alternierende Polarität, 1 Hz, 1 ms, 3.8V (2 mA) konstant, 3h ON/3h OFF	Start postoperativ für 72 Stunden	keine signifikanten Unterschiede im VAS	keine signifikanten Unterschiede beim Analgetikabedarf	k.A.

Tsang et al. 2011 [74]	postoperativer Schmerz (Hysterektomie)	tVNS: 2 x Fossa triangularis, Crus antihelicis, Helix, Antitragus<br>vs.<br>Sham-tVNS: Ohrläppchen, bilateral<br>vs.<br>keine Intervention: Bettruhe	*n=48*<br><br>tVNS=16 (0)<br>Sham-tVNS=16 (0)<br>Kontrolle=16 (0)	IC-4107 (ITO)<br><br>Rechteck, biphasisch, 1 Hz, 0.05-0.15 ms, Amplitude individuell eingestellt	bilateral jeder Stimulationspunkt für je 90 Sekunden	signifikante Reduktion bei ***VAS*** tVNS ggb. Ausgangswert ($p<0.05$) und tVNS vs. Kontrolle ($p<0.05$)<br><br>keine signifikanten Verbesserungen bei PEFR	k.A.	keine
Chakravarthy et al. 2019 [14]	postoperativer Schmerz (Sectio)	pVNS: Antitragus, Crus antihelicis, Fossa triangularis<br>vs.<br>Standard-therapie	*n=100*<br><br>pVNS=47 (3)<br>Kontrolle=49 (1)	ANSiStim (DyAnsys)<br><br>Rechteck, monophasisch, alternierende Polarität, 1Hz, 1 ms, 3.8V (2mA) konstant, 3h ON/3h OFF	Start etwa 1 Stunde nach Eingriff für 48 Stunden	***NRS***-Schmerzintensität signifikant niedriger pVNS vs. Kontrolle<br><br>geringerer Bedarf an Schmerzmedikation in der pVNS-Gruppe vs. Kontrolle<br><br>37 von 50 Patient*innen bewerteten die Verträglichkeit als gut, 12 als akzeptabel, 1 als nicht akzeptabel	k.A.	Anlegen des Geräts nicht toleriert (n=1)
Ahmed et al. 2021 [1]	postoperativer Schmerz (Roux-en-Y Magen-Bypass)	pVNS: Tragus, Scapha, Helix (posterior), Ohrläppchen (Referenzelektrode)<br>vs.<br>historische Kontrollgruppe (Standardtherapie)	*n=18*<br><br>pVNS=8 (0)<br>Kontrolle=10 (0)	NSS-2 BRIDGE (NBD; Innovative Health Solutions)<br><br>Rechteck, 1/10 Hz alternierend alle 2s, 1 ms, 3.2V konstant, 2h ON/2h OFF	Start nach Eingriff für 5 Tage (120h)	60.2% Reduktion in orale Morphinäquivalentdosis postoperativ in den ersten 24h pVNS vs. Kontrolle ($p=0.063$)<br><br>keine Unterschiede bei Nicht-Opiat-Analgetika	28% Schmerzreduktion ($p=0.1$) 24h postoperativ pVNS vs. Kontrolle<br><br>keine Unterschiede bei PONV<br><br>88% (n=7) Patient*innen bewerteten die	k.A.

							Verträglichkeit als exzellent	
Blank et al. 2021 [7]	postoperativer Schmerz (kolorektale operative Eingriffe)	pVNS: 2 x Crus helicis, Helix (posterior), Ohrläppchen (Referenzelektrode)<br>vs.<br>Sham-pVNS: inaktives Gerät	*n=54*<br><br>pVNS=28 (0)<br>Sham-pVNS=24 (2)	NSS (EAD, MFS, BRIDGE; Innovative Health Solutions)<br><br>Rechteck, 1/10 Hz alternierend alle 2s, 1 ms, 3.2V konstant, 2h ON/2h OFF	Start kurz vor Eingriff für 5 Tage (120 Stunden)	kein Unterschied bei oraler Morphinäquivalentdosis pVNS vs. Sham-pVNS<br><br>Subgruppenanalyse: Vorteil für Patient*innen nach ***offenem Eingriff***, signifikant geringere ***orale Morphinäquivalentdosis*** (p = 0.0278), ebenso bei Patient*innen 60-70 und >70 Jahre im Vergleich zu Altersgruppen 30-40, 40-50, 50-60 (p = 0.01092)	generelle Abnahme im STAI<br><br>keine signifikanten Unterschiede bei Übelkeit<br><br>***reduzierte Schmerzen NRS*** an Tag 3 nach OP pVNS vs. Sham-pVNS (p = 0.0340)<br><br>generelle Zufriedenheit mit pVNS-Anwendung (8.2 ± 2.6 von 10)<br><br>Zeitpunkt Spitalsentlassung vergleichbar	hartnäckige Übelkeit (pVNS/Sham-pVNS n=2/3)

Chelly et al. 2022 [15]	postoperativer Schmerz (Nierenspende)	pVNS: Tragus, Scapha, Helix (posterior), Ohrläppchen (Referenzelektrode), links oder rechts<br>vs.<br>Standardtherapie	*n=20*<br><br>pVNS=10 (0)<br>Kontrolle=10 (0)	NSS-2-BRIDGE (Innovative Health Solutions)<br><br>Rechteck, 1/10 Hz alternierend alle 2s, 1 ms, 3.2V konstant, 2h ON/2h OFF	Start nach Eingriff für 5 Tage (120 Stunden)<br><br>Follow-Up für 7 Tage	pVNS bewirkte eine 75.4% Reduktion der ***oralen Morphinäquivalentdosis*** (p=0.03) 24h postoperativ pVNS vs. Kontrolle	57% ***Schmerzreduktion NRS*** (p<0.05) nach 24h und 73.3% Schmerzreduktion nach 48h (p=0.0004) pVNS vs. Kontrolle<br><br>kein Unterschied bei Einnahme von nicht-Opioid-Analgetika<br><br>Trend von geringerer PONV mit Reduktion von benötigten Ondansetron pVNS vs. Kontrolle<br><br>50% ***frühere Überstellung*** auf Ambulanz/Station (p=0.02) pVNS vs. Kontrolle<br><br>Zeitpunkt Spitalsentlassung vergleichbar<br><br>Behandlungstoleranz pVNS (n=9) wurde als exzellent (78%), gut (11%) und annehmbar (11%) bewertet	keine

**akuter/experimenteller Schmerz**								
Busch et al. 2013 [11]	akuter Schmerz (experimenteller Hitzeschmerz)<br><br>gesunde Proband*innen	tVNS: Conchae, links<br>vs.<br>Sham-tVNS: inaktives Gerät	*n=48*<br><br>tVNS/Sham-tVNS=48 (0)	STV02 (Cerbomed)<br><br>Rechteck, monophasisch, 25 Hz, 0.250 ms, 0.25 – 10 mA (individuell)	2 Einheiten zu je 1 h	erhöhte Schmerzschwelle bei mechanischem und Druckschmerz tVNS vs. Sham-tVNS<br><br>signifikant ***reduzierte Schmerzen*** bei tonischen Hitzereizen über 5 min.	keine relevanten Änderungen bei Atem- oder Herzaktivität<br><br>keine klinisch relevanten Nebenwirkungen während tVNS beobachtbar	leichter Schmerz (tVNS/Sham-tVNS n=2/1)<br>Druckgefühl (tVNS/Sham-tVNS n=8/6)<br>Prickeln (tVNS/Sham-tVNS n=12/2)<br>Jucken (tVNS/Sham-tVNS n=10/1)<br>Kitzeln (tVNS/Sham-tVNS n=7/2)<br>merkwürdiges Gefühl (tVNS/Sham-tVNS n=1/0)<br>Schluckbeschwerden (tVNS/Sham-tVNS n=1/0)
Laqua et al. 2014 [44]	akuter Schmerz (experimentelle Schmerzschwelle)<br><br>gesunde Proband*innen	tVNS: Cavum Conchae, bilateral, Referenzelektrode auf Mastoid Knochen<br>vs.<br>Sham-tVNS: inaktives Gerät	*n=22*<br><br>tVNS/Sham-tVNS=21 (1)	TNS SM 2MF (Schwa-medico)<br><br>Rechteck, biphasisch, Burstmodus, 2/100 Hz alternierend, 0.2 ms, maximal tolerierbare Amplitude	2 Studieneinheiten je 35 Min<br><br>5 Schmerzschwellwert-messungen/Einheit (Ausgangswert vor Stimulation, 15/30/40/60 min nach Stimulationsstart)	keine Unterschiede bei Schmerzschwellenwerten (PT) zwischen den Einheiten<br><br>15 Proband*innen reagierten mit einem ***erhöhter Schmerzschwelle*** tVNS vs. Sham (p<0.01)	Herzrate und Blutdruck blieben unverändert	Synkope aufgrund von Bradikardie und Hypotonie (tVNS/Sham-tVNS n=1/0)

						weitere 6 Proband*innen berichteten von ***herabgesetzter Schmerzschwelle*** tVNS vs. Sham ($p<0.05$)		
Frøkjær et al. 2016 [27]	akuter Schmerz, Darmmotilität<br><br>gesunde Proband*innen	tVNS: Cymba Conchae, links<br>vs.<br>Sham-tVNS: Ohrläppchen, links	*n=18*<br><br>tVNS/Sham-tVNS=18 (0)	NEMOS (Cerbomed)<br><br>Rechteck, monophasisch, 30 Hz, 0.25 ms, individuelle Amplitude (0.1-10 mA)	60 min Stimulation mit 2x10 min tiefer langsamer Atemtechnik	tVNS vs. Sham-tVNS erhöht die ***Schmerzschwelle*** gegenüber ***Knochenschmerz*** ($p=0.001$)<br><br>kein signifikanter Effekt bei Muskelschmerz	der kardiale ***vagale Tonus*** stieg während aktiver tVNS vs. Sham-tVNS an ($p=0.009$)	keine
Usichenko et al. 2017 [75]	akuter Schmerz (experimenteller Hitze-Schmerz)<br><br>gesunde Proband*innen	tVNS: Conchae, bilateral<br>vs.<br>Sham-tVNS: inaktives Gerät	*n=20*<br><br>tVNS/Sham-tVNS=20 (0)	TENS Gerät (Eigenkonstruktion)<br><br>Rechteck, 8 Hz, 0.2 ms, individuell deutlich wahrnehmbare Amplitude (~7.6mA)	45 Min/Einheit	in der Gesamtanalyse kein Effekt auf Schmerzschwelle<br><br>Untergruppenanalyse zeigte bei 8 Proband*innen signifikant ***erhöhte Schmerzgrenze*** nach tVNS vs. Sham-tVNS ($p=0.024$)<br><br>12 Proband*innen signifikant ***erniedrigte Schmerzgrenze*** tVNS vs. Sham-tVNS ($p=0.012$)	8 Responder zeigten während tVNS ein schwächeres fMRI-Signal im rechten Caudate Nucleus, dem vmPFC, der linken anterioren Insel, und dem linken Hypothalamus im Vergleich zu Sham-tVNS	k.A.

Janner et al. 2018 [33]	akuter Schmerz (experimenteller Hitze-Schmerz)<br><br>gesunde Proband*innen	keine Intervention<br>vs.<br>tVNS: <u>Cymba Conchae</u>, links und rechts<br>vs.<br>Sham-tVNS 1: Ohrläppchen<br>vs.<br>Sham-tVNS 2: <u>Cymba Conchae</u>, inaktives Gerät	*n=51*<br><br>keine Intervention/tVNS/Sham-tVNS 1/Sham-tVNS 2=49 (2)	PuntoBravo (Medizintechnik Rostock)<br><br>Rechteck, Blöcke zu 9 Impulsen, 100 Hz, 0.2 ms, 2x/Sek (gemischtes Frequenzmuster, 100Hz/ 2Hz)	Start 20 Min vor Beginn des Hitzereizes je 25 Min/Einheit<br><br>repetitive Hitzestimulation	***Schmerzintensität VAS*** war geringer in allen Interventionsgruppen im Vergleich zur Gruppe ohne Intervention (p<0.001)<br><br>hypoalgetischer Effekt/Effekt auf TSP zeigte geschlechtsspezifische Unterschiede	Individuelle Schmerztoleranz-Temperaturen und STAI-Werte waren vergleichbar unter allen experimentellen Bedingungen	k.A.
Farmer et al. 2020 [22]	akuter Schmerz (ösophagaler Schmerz Hypersensibilität)<br><br>gesunde Proband*innen	tVNS: <u>Cymba Conchae</u>, links<br>vs.<br>Sham-tVNS: Ohrläppchen, links	*Exp. 1: n_1=34* (19*)<br>tVNS/Sham-tVNS=15 (0)<br><br>*Exp. 2: n_2=25* (7*)<br>tVNS/Sham-tVNS=18 (0)<br><br>*Non-Sensitizers wurden nach Klassifizierung ausgeschlossen	NEMOS (Cerbomed)<br><br>Rechteck, 25 Hz, 0.1 ms, mA individuell auf deutliche, Wahrnehmbarkeit, 30s ON/30s OFF	25 Min/Einheit<br><br>Exp.1: Stimulation während Säureinfusion<br><br>Exp.2: Stimulation nach Säureinfusion	Exp.1: tVNS ***verhindert*** die Entstehung von säureinduzierter ösophagaler ***Hypersensibilität*** (p=0.004) vs. Sham-tVNS<br><br>Exp. 2: tVNS ***reversiert*** die säureinduzierte ösophagale ***Schmerz-Hypersensibilität*** (p=0.0001) vs. Sham-pVNS	Exp. 1: ***kardialer vagaler Tonus*** Anstieg tVNS vs. Sham-tVNS (p=0.016)<br><br>Exp. 2: ***kardialer vagaler Tonus*** Anstieg tVNS vs Sham-tVNS (p = 0.03)	k.A.
Dumoulin et al. 2021 [19]	akuter experimenteller Schmerz<br><br>gesunde Proband*innen	tVNS: <u>Cymba Conchae</u>, links<br>vs.<br>Sham-tVNS: Ohrläppchen, links	*Exp. 1: n_1=22*<br>tVNS/Sham-tVNS=22<br><br>*Exp. 2: n_2=15*<br>tVNS/Sham-tVNS=15	NEMOS (Cerbomed)<br><br>Rechteck, monophasisch, 25 Hz, 0.25 ms, Amplitude individuell auf deutliche Wahrnehmbarkeit, 30s ON /30s OFF	3h (Exp.1) bzw. 1h (Exp.2)	weder tVNS noch Sham-tVNS zeigten einen Effekt auf zerebrale und Verhaltensaspekte von somatosensorischer Perzeption	k.A.	k.A.

[a] Abkürzungen: ADL – Activities of Daily Living; BPI – Brief Pain Inventory; CSBM – Complete Spontaneous Bowel Movements; DAS28-CRP – Disease Activity Score-28 with C-Reactive Protein; FDI – Functional Disability Inventory; FIHOA - Functional Index for Hand Osteoarthritis; FU – Follow-up; HAQ-DI – Health Assessment Questionnaire Disability Index; HIT-6 – Headache Impact Test; IBS-SSS – Irritable Bowel Syndrome-Severity Scoring System; ITT – Intention-To-Treat; MIDAS - Migraine Disability Assessment; mPFC – Medial Prefrontal Cortex; MSQ – Migraine-specific QoL Questionnaire; NRS – Numeric Rating Scale; PANAS – Positive and Negative Affect Scale; PEFR – Peak Expiratory Flow Rate; PFSD – Pain Frequency-Severity-Duration; PGA – Physician Global Assessment; PoCG – postcentral gyrus; PONV – post-operative Übelkeit und Erbrechen; PP – per Protokoll; PtGA – patient global assessment; PSS – perceived stress scale; SAS/SDS – self-rating anxiety scale/self-rating depression scale; pVNS – perkutane Vagusnervstimulation; SCL –skin conductance level; SFG – superior frontal gyrus; STAI – State-Trait Anxiety Inventory; STAI-C – State-Trait Anxiety Inventory for Children; TENS – transkutane elektrische Nervenstimulation; tVNS – transkutane Vagusnervstimulation; VAS – visuelle Analogskala; WCC – ways of coping checklist.

[b] Unterstrichene Lokalisationen im Ohr in der Methode kennzeichnen vagal innervierte Bereich.

[c] Fett hervorgehobene Textteile in den primären & sekundären Ergebnissen kennzeichnen statistisch signifikante Veränderungen.
